# EEG-IP: an international infant EEG data integration platform for the study of risk and resilience in autism and related conditions

**DOI:** 10.1186/s10020-020-00149-3

**Published:** 2020-05-07

**Authors:** Stefon van Noordt, James A. Desjardins, Scott Huberty, Lina Abou-Abbas, Sara Jane Webb, April R. Levin, Sidney J. Segalowitz, Alan C. Evans, Mayada Elsabbagh

**Affiliations:** 1 Montreal Neurological Institute, Azrieli Centre for Autism Research, McGill University, Montréal, Canada; 2 Compute Ontario, St. Catharines, Canada; 3 McGill University Health Centre, Montréal, Canada; 4 Center on Child Health, Behavior and Development, Washington Children’s Research Institute, Washington, WA USA; 5 Boston Children’s Hospital, Boston, MA USA; 6 Cognitive and Affective Neuroscience Lab, Brock University, St. Catharines, ON Canada; 7 McConnell Brain Imaging Centre, McGill Univeristy, Montréal, Canada

**Keywords:** EEG, Autism risk, Biomarkers, Pre-processing, High performance computing, ICA

## Abstract

**Background:**

Establishing reliable predictive and diganostic biomarkers of autism would enhance early identification and facilitate targeted intervention during periods of greatest plasticity in early brain development. High impact research on biomarkers is currently limited by relatively small sample sizes and the complexity of the autism phenotype.

**Methods:**

EEG-IP is an International Infant EEG Data Integration Platform developed to advance biomarker discovery by enhancing the large scale integration of multi-site data. Currently, this is the largest multi-site standardized dataset of infant EEG data.

**Results:**

First, multi-site data from longitudinal cohort studies of infants at risk for autism was pooled in a common repository with 1382 EEG longitudinal recordings, linked behavioral data, from 432 infants between 3- to 36-months of age. Second, to address challenges of limited comparability across independent recordings, EEG-IP applied the Brain Imaging Data Structure (BIDS)-EEG standard, resulting in a harmonized, extendable, and integrated data state. Finally, the pooled and harmonized raw data was preprocessed using a common signal processing pipeline that maximizes signal isolation and minimizes data reduction. With EEG-IP, we produced a fully standardized data set, of the pooled, harmonized, and pre-processed EEG data from multiple sites.

**Conclusions:**

Implementing these integrated solutions for the first time with infant data has demonstrated success and challenges in generating a standardized multi-site data state. The challenges relate to annotation of signal sources, time, and ICA analysis during pre-processing. A number of future opportunities also emerge, including validation of analytic pipelines that can replicate existing findings and/or test novel hypotheses.

## Introduction

Biomarkers for autism would substantially advance early identification and intervention, improving oucomes for individuals with the disorder, and thus reducing the burden of autism to these individuals and their communities. Neuroimaging cohort studies of infants at risk for autism are illuminating altered early brain development that underlies autism prior to the onset of behavioral symptoms, providing opportunities to identify periods of greatest plasticity and responsiveness to treatment. Infants are typically considered at elevated risk because they have an older sibling who has been diagnosed with ASD. To date, infant sibling neuroimaging studies have revealed indicators of risk that relate to later diagnosis, emerging atypicality in brain development, and concurrent and/or longitudinal functional outcomes.

For example, several studies have revealed that early infant autism risk is associated with mechanisms of visual attention and that at-risk infants have distinct neural responses to social stimuli, including faces and dynamic gaze patterns (Elsabbagh et al. 2009; Lloyd-Fox et al. 2013; Orekhova et al. 2014), attentional disengagement (Elsabbagh et al. 2013), and auditory language processing (Riva et al. 2018), which relate to autism diagnosis in later life (Elsabbagh et al. 2012). These differences occur in multiple modalities, suggesting that ASD is characterized, in part, by alterations in functional integration across neural networks. Other research has shown atypical brain development trajectories in infants who are at risk for autism with or without a diagnosis. Such risk indicators have been documented in relation to resting EEG power (Tierney et al. 2012), functional connectivity (Orekhova et al. 2014; Righi et al. 2014), and measures of EEG time series complexity (Bosl et al. 2018; Bosl et al. 2011). In addition to indicators of risk and emerging atypicality, some evidence suggests that EEG measures also relate to functional outcomes, such as quality of parent-infant interactions (Elsabbagh et al. 2014) and language development (Levin et al. 2017).

Despite the rapid progress and value of the findings in illuminating neural mechanisms of risk, research progress on infant EEG markers of autism is hampered by several factors. First, there is considerable cost, both in time and financial investment, to acquiring infant EEG data from populations at risk for neurodevelopmental disorders, which limits the opportunities for independent replication of discovered biomarkers. Further, no study has yet tested the value of a putative marker in a large, population-representative sample. Second, independent samples collected from different laboratories only offer modest statistical power to detect effects, especially in the group that goes on to develop autism later in life (typically 20% of at-risk infants; Ozonoff et al. 2011). Third, variation in a number of methodological factors relating to samples, paradigms, and signal processing has also contributed to inconclusive results in EEG studies, both with infants at risk as well as older diagnosed children and adults (O’Reilly et al. 2017).

Some sources of variation relate to the intrinsic heterogeneity of ASD, which is increasingly becoming the focus of recent theoretical models of etiology and development of the condition (Elsabbagh & Johnson 2016). Other factors, namely methodological ones, are possible to address. A recent systematic review of EEG findings in autism across the lifespan has demonstrated that in spite of substantial variation in results across studies, there was strong support for a pattern of long range underconnectivity (O’Reilly et al. 2017). The review also synthesized the factors currently limiting comparability across studies with respect to identifying other patterns of brain connectivity. These factors include differences in sample characteristics (e.g., age, symptom severity, IQ), experimental paradigms (e.g., tasks parameters, stimuli, control conditions), data acquisition parameters (e.g., hardware, testing environment), and EEG signal extraction (pre-processing pipelines, quality control, statistical models).

Some of these challenges have been recognized for a long time, with complementary efforts to address them. In recent years, consensus has been developed around EEG data acquisition and signal processing in autism, in an effort to increase validity and reliability of findings from independent studies (Webb et al. 2015). Further, new signal processing pipelines are being developed to enhance signal-to-noise ratios, especially for data from infants and atypical populations where data is relatively more influenced by artifacts (Gabard-Durnam et al. 2018; Levin et al. 2018; Zima et al. 2012). Finally, a major push towards an Open Science framework in neuroscience in general (Ali-Khan et al. 2019; Das et al. 2016, 2017; Poupon et al. 2017) has led to increased interest in data pooling across independent samples but to date, no such effort has been developed for EEG data in autism. Success in development of data repositories for other neuroimaging data of ASD, namely (f) MRI, has demonstrated feasibility and value of pooled research samples from independent studies (Di Martino et al. 2014). However, these efforts have also raised debate around the importance of going beyond data pooling, towards data standardization to facilitate aggregated data sets that also share methodology in terms of pre-processing and quality control.

To simultaneously address challenges in data pooling and standardization in cohort studies with infant siblings, we established the International Infant EEG Data Integration Platform (EEG-IP). The EEG-IP approach is not limited to infant data and is fully extendable for use with other multi-site EEG datasets. EEG-IP integrates three complementary components for advancing research on infant autism EEG biomarkers: (1) A data repository structure allowing for a centrally pooled data set of independently collected cohorts of infant sibling EEG data; (2) The adoption of the Brain Imaging Data Structure (BIDS; Gorgolewski et al. 2016) extension for EEG (BIDS-EEG; Pernet et al. 2018) that harmonizes the storage of EEG acquisition parameters, as well as experimental and individual difference variables in a common framework across pooled projects; (3) Implementation of the Lossless signal processing pipeline (https://github.com/BUCANL/bids_lossless_eeg) that produces a common EEG data state that both maximizes signal isolation and minimizes data loss by applying quality control measures on each recording session in the data set. The Lossless pipeline performs several data quality assessment procedures, Adaptive Mixture Independent Component Analysis (AMICA), and signal property annotation.

Use of these complementary technical solutions gives rise to a fully standardized data state, with all experimentally relevant information about each of the pooled cohorts is retrievable within a common framework. Further, it provides a unified and standardized output data state in which cortical signal is maximally isolated from the various sources of noise in the EEG data and data loss is minimized. In turn the standardized data state offers maximal data exploration possibilities at large scales on cortical EEG data, substantially accelerating hypothesis testing in biomarker discovery research.

## Methods and materials

Three independent sites (Boston Children’s Hospital, Boston MA; Birkbeck University, London England; University of Washington, Washington WA) contributed data to the EEG-IP platform. Together, this pooled data set consists of 1455 EEG recording sessions (Boston: 972; London: 188; Seattle: 223) from 446 unique participants (Boston: 250; London: 106; Seattle: 90), spanning multiple age ranges (Boston: 3, 6, 9,12, 18, 24, 36 months; London: 7, 14 months; Seattle: 6, 12, 18 months). Many acquisition parameters were common across sites, including use the EGI system with saline nets for EEG data acquisition (EGI, Inc.). A number of recording parameters varied both across and within sites, including electrode montage (129 and 65 channel configurations), task procedures during recording sessions (both event-related response tasks and non-event related data), data annotation (e.g., events contained in the data file describing the experimental context), recording length, file formatting, and signal contamination.

**Fig. 1:**
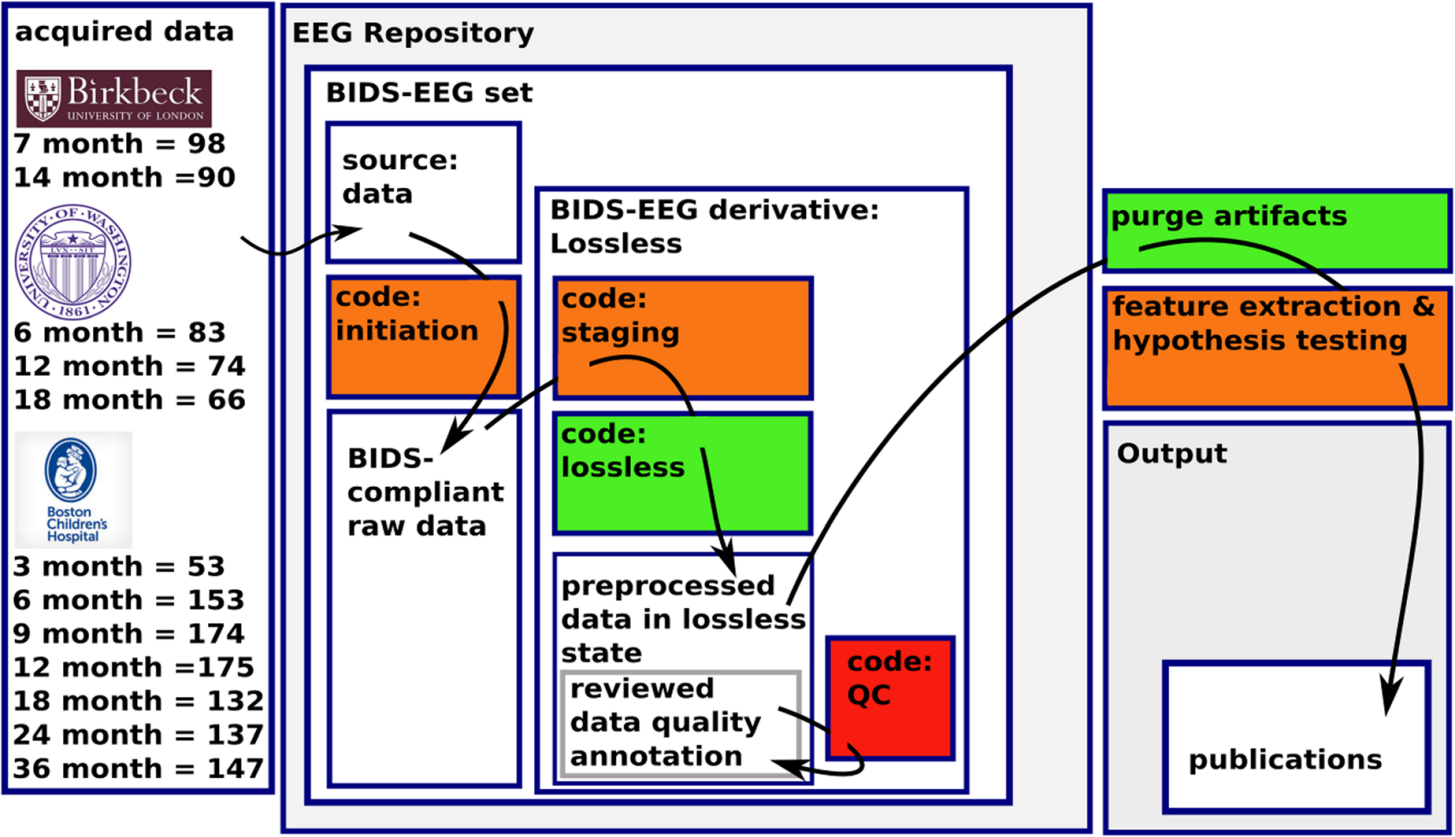
EEG-IP Architecture and Data Flow. Schematic of how “acquired data” flows from the acquisition site through the platform, and ultimately to “publications” output. White boxes represent data states and colored boxes represent coded procedures. Orange boxes represent procedures that may vary from project to project often handling the harmonization of unique data set features. The green box represents the maximally standardized process that is designed to run similarly across projects and is optimized for execution on HPC resources. The red box is the manual process in which each file is inspected and annotated by a reviewer. “Acquired data” enters the platform as unmodified raw data files in the “BIDS-EEG set” as “Source data”. The “source data” is then processed with the “initiation” to become “BIDS-compliant raw data” (open file format and containing the appropriate annotation files, etc.). Once the data are BIDS-compliant they are processed into the “BIDS-derivative Lossless” state beginning with a “staging” procedure designed to harmonize properties of the data (e.g., coregister channel locations to a standard head surface) then run through the “Lossless” pipeline. Each recording session file in the resulting “preprocessed data in Lossless state” is then examined by a reviewer in an optimized QC procedure that results in “reviewed data quality annotation”. From this pre-processed state the annotations are used to guide the post processing “feature extraction & hypothesis testing” procedures that result in the output “publications”

The strategy by which the EEG data from the various sites are processed into a single compatible state is illustrated in Fig. 1, and described below. This diagram illustrates how data move from the acquisition site into a BIDS-EEG compliant format. The BIDS-EEG compliant data files are then processed for signal isolation via the Lossless pipeline and interactive quality control, producing the final Lossless data state that is ready for post-processing and hypothesis testing.

In Fig. 1, the orange boxes indicate procedures that may be unique to each of the pooled projects. For example, preceding the execution of the Lossless pipeline, each acquisition project may require a unique process to standardize the raw data into a BIDS-EEG compliant format (e.g., this is often the case for task event codes that require Hierarchical Event Descriptor [HED] translations) Within the Lossless pipeline, data staging may be required (e.g., transforming the channel coordinate montage to conform to a common head surface, notch filtering of different line noise frequencies, etc.). Following the execution of the Lossless pipeline, unique post-processing scripts may be applied. Although each of these procedures may be unique to the specific acquisition or post-processing project, once the process is established for that project it may be executed in a fully automated manner. The red box, “code: QC”, indicates a process that requires manual interaction on each file.

The goal of the Lossless pipeline within EEG-IP is not to fully automate the signal processing, but rather to isolate manual interaction to a single step that is optimized, with flexible figure interactions, and provides sufficient data classification information for the reviewer to make informed decisions about what data portions should remain for hypothesis testing. The green boxes represent procedures that are fully automated and executed in an unsupervised manner compatible with high performance computer scheduling. Although the fully automated procedures are configurable by several parameters, the optimal parameter settings are designed to be highly transferable across data sets. For a detailed description of the methods applied in the staging procedure of the EEG-IP set see Supplementary Materials.

## Results

In what follows, we compare performance of EEG-IP in standardization across key parameters where there was variation, namely time, channels, and IC analysis.

### Time annotation

**Fig. 2:**
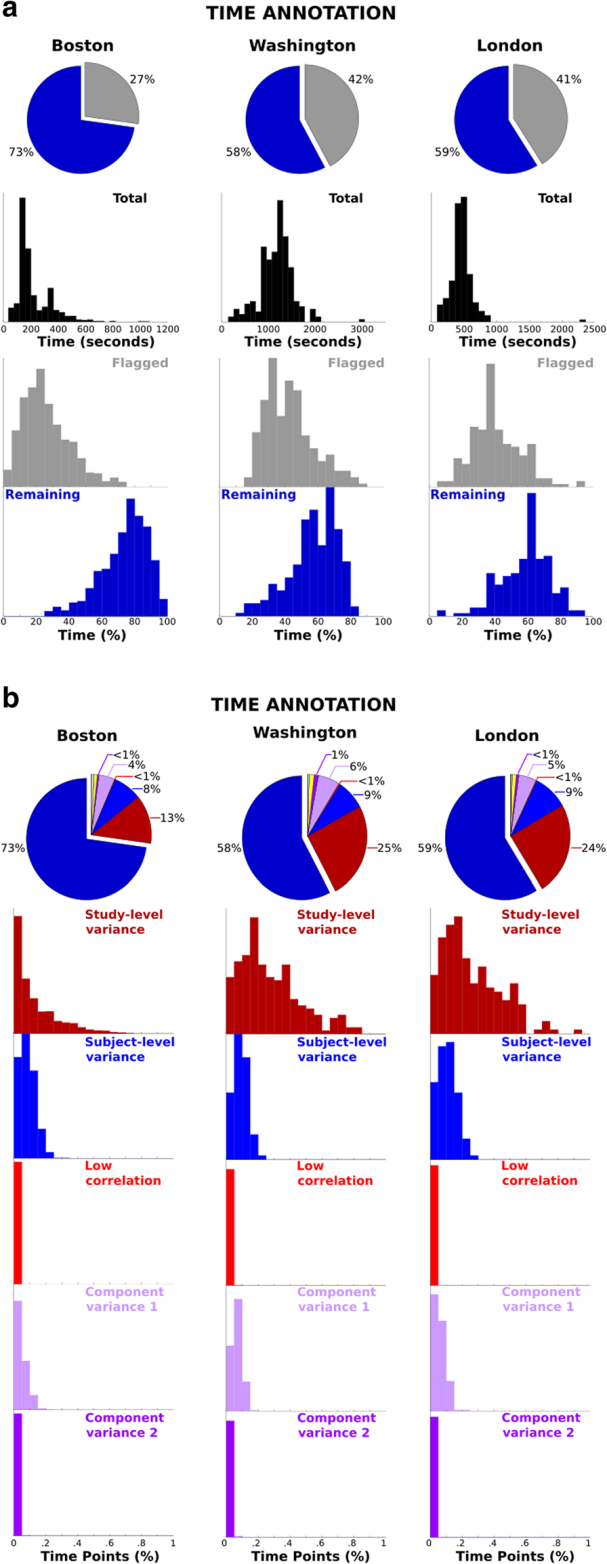
Time annotation. **a.** Pie charts show average proportion of time points (seconds) remaining (dark blue) and flagged (grey) for removal following the Lossless pipeline. Histograms show distribution of total (black), flagged (grey), and remaining (blue) time points across the three sites. **b.** Pie charts show average proportion of time points flagged for removal due to study-level staging voltage variance (dark red), individual subject-level voltage variance (blue), low correlation between neighboring channels (red), component activation variance from first ICA (magenta), and component activation variance from second ICA (purple). The other slices reflect non-task time marked due to gaps between events (e.g., task lead up, task breaks, etc). Histograms show distribution of proportion of time points in the various categories across three sites

In EEG recording, there are typically periods of time that are not recoverable for use in hypothesis testing. Various types of artifacts could be addressed by data removal or transformation (see Bigdely-Shamlo et al. 2015; Gabard-Durnam et al. 2018). The Lossless pipeline maintained as much spatially stationary (scalp signals made up of field projections emanating from spatially fixed sources) time as possible with the goal that the stationary artifacts (e.g., eye blinks, EMG, etc) can be isolated by ICs. The pie charts in Fig. 2a depict the average proportion of time points remaining (dark blue) and flagged for removal (grey) following the Lossless pipeline. The blue histograms depict the number of seconds remaining for each of the files from the sites and the grey histograms represent the percentage of removed time for each of the recordings from the site. Although each of the sites have a different distribution of the recording times (Boston is the shortest followed by London and Washington) the distributions of time point proportions removed is similar across sites. Regardless of these task recording differences, the relative distribution of time flagged is similar across sites. This similarity is also reflected in the distribution of flagging based on different data properties.

Beyond the global retained versus removed time periods, the plots in Fig. 2b describe the specific measures that resulted in the removal of time periods. The pie graphs represent the percentages of total times removed for specific data properties: study-level staging voltage variance (“*ch_s_sd*”, dark red), subject-level relative voltage variance (“*ch_sd*”, blue), low neighbor correlations (“*low_r*”, red), initial IC projection voltage variance (“*ic_sd1*”, magenta) and second IC projection voltage variance (“*ic_sd2*”, purple). In these figures, the three sites are handled similarly by the pipeline, such that a large majority of the time removed is classified by the staging activation determination based on the pool level parameter estimate (i.e., “*ch_s_sd*”). This is an important property of the pipeline because it indicates that the processing is able to handle large percentages of data in a recording being contaminated with large artifacts, but still progressively be sensitive to fine grained distinctions of non-stationarity artifacts. In the histograms, we see that 80–90% of some of the files are flagged during the staging criteria. We also note that the Boston site has the least staging criteria rejection (as well as the least total rejected time-periods) which may relate to the selection of shorter recording periods and the inclusion of only resting state data. Following the staging criteria, the subsequent measures are extremely similar across each site, having averages within 1–2% in terms of relative percentage of time flagged with “*ch_sd*” and “*ic_sd1*” accounting for the majority of the within recording criteria and “*low_r*” and “*ic_sd2*” capturing properties of the data that constitute sublte artifacts and spatial non-stationarity, which are distinct from large voltage fluctions.

### Channel annotation

**Fig. 3:**
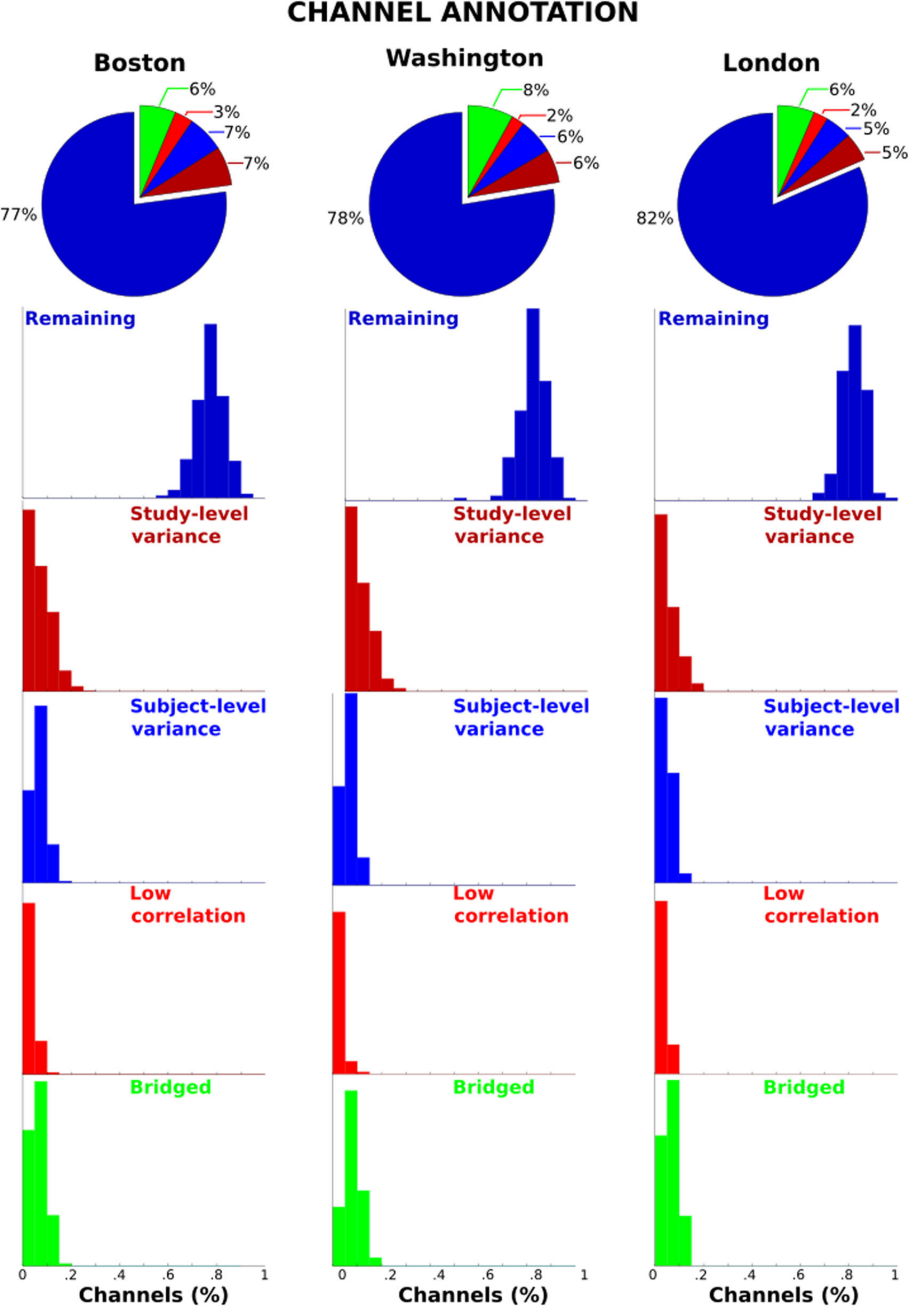
Channel annotation. Pie charts show average proportion of channels retained (dark blue) and those flagged for removal due to study-level staging voltage variance (dark red), individual subject-level voltage variance (blue), low correlation between neighbouring channels (red), and bridging between neighbouring channels (green). Histograms show distribution of proportion of channels in the various categories across three sites

In regards to channel selection the three sites also have very similar outcomes such that the average channel retention for the three sites is between 77 and 82%. The histograms in Fig. 3 show that the distribution of percentage of channels retained (dark blue) is clustered around the group mean (whether 65 or 129 channel montage). Further, the relative distributions of the channels classifications in “*ch_s_sd*”, “*ch_sd*”, “*bridge*” and “*low_r*” are similar across sites, with differences of the averages at only 1–2%.

### IC annotation

**Fig. 4:**
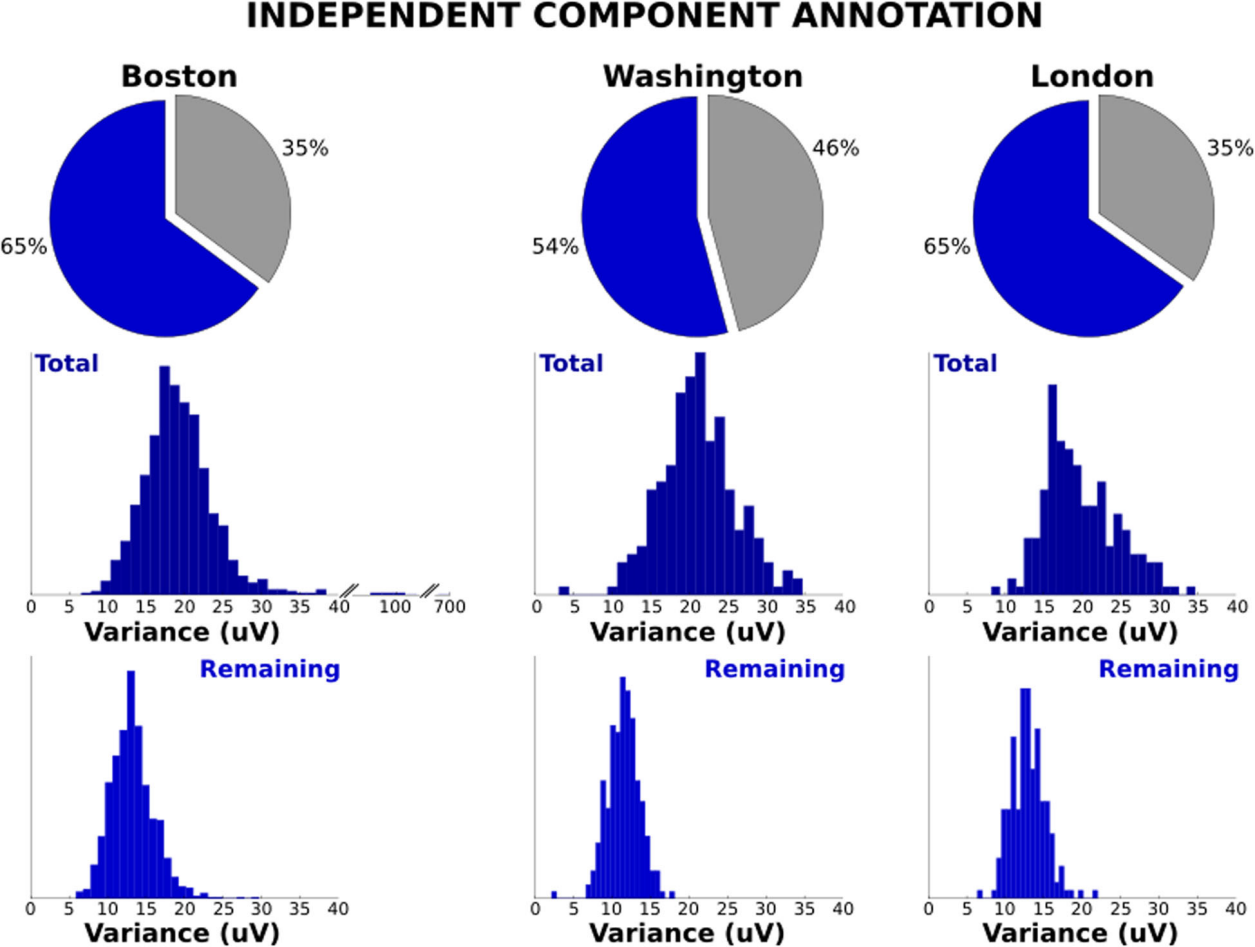
Component annotation. Pie charts show average proportion of EEG signal variance accounted for by components that were retained (dark blue) and those flagged for removal (grey). Histograms show distribution of signal variance in total scalp EEG signal (dark blue) and retained EEG signal (blue) following the removal of flagged components across the three sites

Once the time points and channels have been flagged for various reasons, it is by design in the Lossless pipeline that stationary artifacts remain in the unflagged channels and time periods. The spatially stationary artifacts are isolated by ICs and flagged for removal. The IC pie charts in Fig. 4 depict the average spatial variance (standard deviation across scalp channels at each time point) for the remaining ICs (non-flagged ICs projected back to the scalp) and the average spatial variance of the ICs flagged for rejection. Again, the three sites show similar patterns indicating that more than 50% of the spatial variance in the remaining channels and time points are classified as consisting of non-artifactual ICs. The remaining IC pro jections show similar amplitude characteristics as the distribution of spatial variance across subjects centers around 10 µV for each site. These diagnostic properties clearly show that, despite different sources of variance that may contribute to non-stationary artifacts (e.g., acquisition parameters, paradigms, individual differences across subjects), the Lossless pipeline is capable of isolating similar signal properties across cohorts which correspond to cortical activation properties.

### Power spectrum profile

**Fig. 5:**
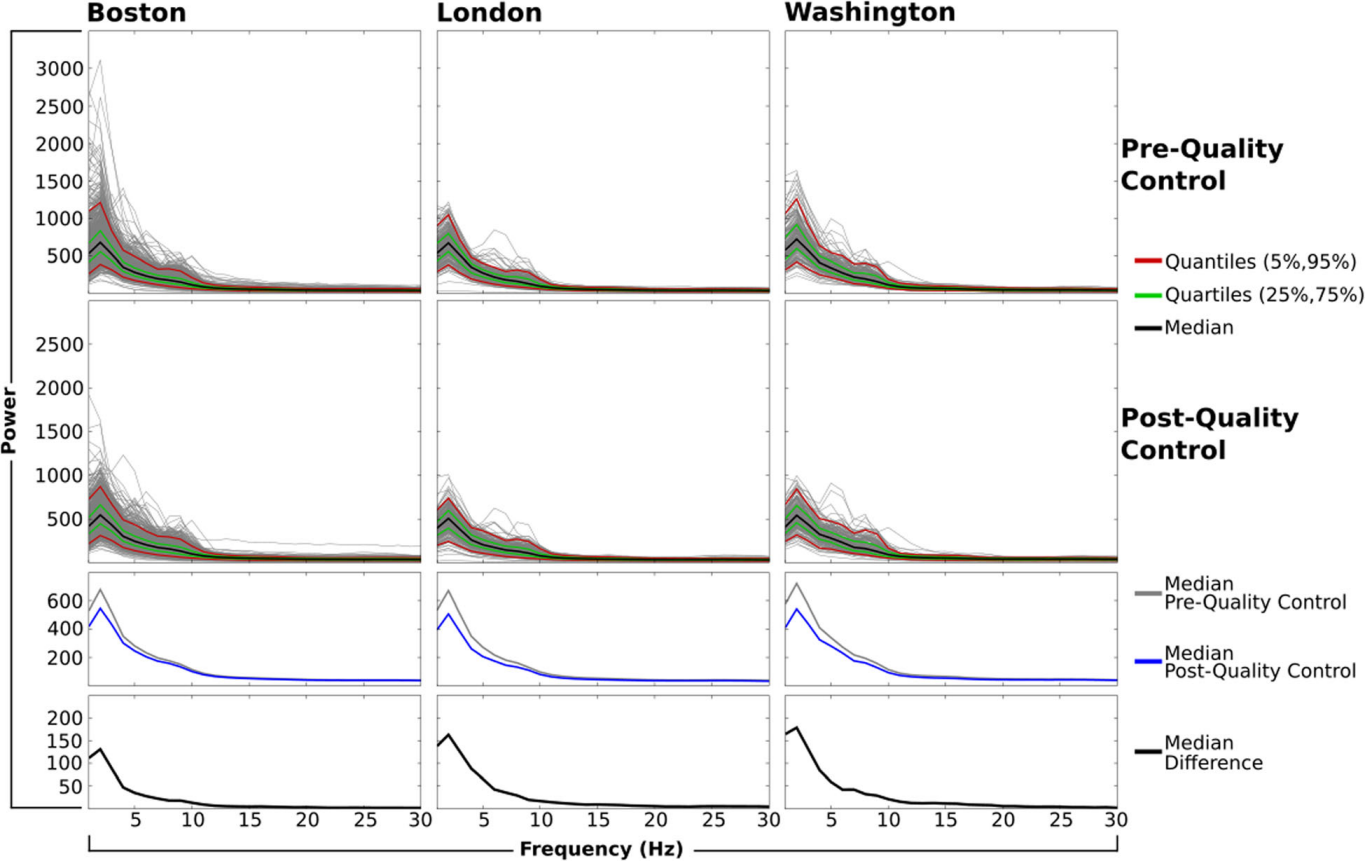
Power spectrum profile at channel Cz pre and post-quality control. Spectral power (1–30 Hz) profile pre-quality control (top) and postquality control once flagged time, channels, and ICs were removed (middle). Lines represent single subjects (grey), median (black), quartiles (green), and quantiles (red). For comparison across sites, median spectral power is plotted separately for pre (grey) and post-quality (blue), as well as the median difference between pre and post-quality control (black, bottom)

The raw power spectrum profile from central channel Cz is shown for each site in Fig. 5. This summary shows that the pipeline and quality control review process result in a similar power spectrum profile of the data across cohorts that are retained for post-processing and hypothesis testing.

## Discussion

Identifying early biomarkers of autism is critical given that the first years of life represent greatest plasticity, when infants may be most responsive to treatment that can mitigate the long-term severity of the disorder. Relative to other neuroimaging methods such as MRI and MEG, EEG is less invasive, more cost effective, and superior in its temporal resolution, making it a feasible technology that can be implemented, on a large scale, in research labs across the globe. EEG methodology has sparked major progress in developmental neuroscience (Jeste et al. 2015) and is the best-established method for investigating neural processes in infants, making it well suited for detecting early biomarkers of autism before the emergence of behavioral symptoms.

However, this valuable line of research is currently limited by the fact that EEG collection in infants and populations with neurodevelopmental disorders introduces issues related to recording duration and signal quality. Independent studies with EEG have been limited by relatively small samples (O’Reilly et al. 2017), which have reduced power to detect statistical differences in the subgroup of infants who go on to develop autism, introducing risks of both Type I (false positive) and Type II (false negative) errors. Large sample sizes that are combined across studies are needed to provide enhanced analytic and statistical power sufficient to identify and validate early biomarkers. However, previous attempts to pool independent samples in neuroimaging data, namely MRI, has resulted in mixed findings (Traut et al. 2018), highlighting the need to establish standardized data states of pooled data that are of high quality. Another challenge for infant research is the reduced signal-to-noise ratio due to more artifacts in infants those with neurodevelopmental disorders. Further, to date, there have been limited application of high performance computing methods in order to standardize large scale pooled data across independent samples.

EEG-IP was developed to simultaneously address the need for data pooling as well as comparability across independent samples, by generating an integrated standardized data state, using a combination of High Performance Computing (HPC) solutions. This included use of EEGLAB data structure file format within the BIDS-EEG standard, which together maintain all of the uniqueness of each project’s acquisition parameters within their defined metadata files (Pernet et al. 2018). Specifically, critical acquisition parameters such as sampling rate, channel locations, experimental control event marking, filtering, etc., are stored in a manner that is universal regardless of the employed acquisition documentation practices.

EEG-IP also used an open source signal processing tool, the Lossless pipeline to standardize signal properties. EEG pre-processing pipelines are generally sensitive to various aspects of the data acquisition parameters, such as the number of scalp sites, recording duration, environmental noise sources, participant behaviour, and reduced signal-to-noise ratio in data from infants and/or populations with neurodevelopmental disorders (Levin et al. 2018; Zima et al. 2012). The performance of pre-processing procedures are further impacted by the increased artifact contamination in infant EEG. We selected the Lossless pipeline because it offers comprehensive quality control assessment, alongside expandable tools to isolate reliable cortical signal from noise in the EEG, maintain maximal information from raw recordings, generalizable parameters, replicable procedures across sites and projects, and batch processing to scale up analyses to efficiently handle hundreds or thousands of datasets using HPC clusters.

While equating important aspects of measurement in the EEG, such as the re-referencing to an average common standard head montage, the Lossless strategy adopted in this platform also has an emphasis on being robust to variations across acquisition projects. By progressing from coarse grained measures of scalp voltage outliers to find grained measures of spatial stationarity, this process is able to both remove large non-stationary artifacts (e.g., substantial movement artifacts, sweat artifacts, etc.) in the data prior to the ICA decomposition and maintain in the data periods of time where spatially stationary artifacts (e.g., heart artifacts, eye movements, blinks, muscle activation, etc.) can be accounted for by isolated ICs.

The application of the Lossless pipeline has highlighted a number of challenges. The first challenge in achieving a reliable ICA decomposition in infant data has to do with the duration of data that is provided to the modeling. By focusing on the classification of non-stationary channels and time points, this strategy maximizes the quantity of useful signal that is available to ICA modelling. This being said, recording duration is still an important factor affecting the quality of the pre-processed data state that can potentially be addressed by modified data acquisition procedures. Moving forward, we recommend that acquisition procedures include as much time as possible in the recording while the subject is relatively motionless, even if there is no experimental task or state in progress at the time. This extra recording time of stationary signal (e.g., a minimum of a few minutes to up to an hour when feasible) could increase the pipeline’s ability to recover cortical signal from periods of time that are otherwise saturated with artifacts.

Another challenge relates to the variation in number of recording channels. Although dense array montages are readily available, this challenge may remain when integrating more diverse samples and those collected from clinical settings into the platform. Although the process is flexible to the locations in the recording montage, the ICA modelling needs to be provided with enough channels to account for the number of unique field potentials that are recorded from the scalp surface. In this case either increasing the number of stationary recording channels (e.g., greater than 32 channels), or reducing the number of field potentials being picked up at the recording sites, either by external sources (e.g., nearby unshielded power cords, and other electrical equipment, etc.) or internal sources (e.g., various muscle groups around the neck and face). The best way to decrease the number of internal field sources is by minimizing muscle activity in the forehead and neck areas, a goal that may or may not be achievable in future studies.

Notwithstanding the challenges highlighted by the process of translation from raw to standardized data, our diagnostic analysis shows that equivalent EEG signal can be extracted from independent sites with variable acquisition parameters. Although the sites currently contained in the EEG-IP have various acquisition parameters (e.g., channel montage, sampling rate, experimental environment, and recording times, etc.), the outcome state from each of the sites after time and channel rejection, and then IC artifact removal, were comparable in terms of state measures such as voltage range and spectral power. Importantly the similar states of the signal qualities were achieved in a way that was both independent of recording parameters (e.g., various recording montages were all co-registered and re-referenced to a common head surface) and robust to various levels of signal contamination.

The relatively high degree of success in data retention demonstrates that it is possible to optimize data quality by achieving signal and noise isolation while minimizing both data reduction (e.g., rejecting channels and time points, etc.) and data manipulation (e.g., restrictive filtering and various forms of artifact correction). Minimizing the data removal and manipulation during the pre-processing also minimized the constraints on post-processing analyses. Very little information is lost relative to the original raw recording file because signal quality classification is implemented through data annotation rather than through data rejection. In fact, the data annotations regarding the various aspects of signal quality obtained during the processing of the pipeline can be applied to the original raw recording files in order to maintain all of the original information while gaining important and fine grained signal classification information. This minimal manipulation strategy not only enables for diverse post-processing strategies, but it is also an ideal long-term state of EEG that is optimally compatible with future analytic methods. Therefore, relative to conventional approaches used with relatively small independent samples, the standardized state in EEG-IP is can be seen as a “translated” state of the raw state (containing signal quality annotations) rather than a fully pre-processed state. Furthermore, this data state also makes an ideal starting point for future advancements in pre-processing strategies and post-processing analytics.

## Conclusions

By favoring annotation of the data over its manipulating or pruning, not only is the standardized state of EEG-IP unique for its data retention but, perhaps more notably, it is also novel for the degree of quality control inspection that was applied to each file. Although pre-processing pipelines are becoming very powerful and capable of sophisticated unsupervised and fully automated decision making, this platform focuses on an efficient yet exhaustive interactive quality control review process. In this way the signal classification annotation not only allow for the full retention of data but also allows the quality control reviewer to consider augmented signal quality visualizations when making final decision about the recording channels, time points and ICs. The minimization of the data manipulation and reduction in exchange for data annotation, together with the pipeline’s maximization of the isolation between signal and noise, make this platform uniquely suited for long term and evolving discovery. Further expansion and technical improvements to EEG-IP will expand data access to multiple users for collectively or independently testing targeted hypotheses. Linked curated data is also accessible for future data mining and new analytic tools, such as assessing the robustness of single subject effects to unpack phenotypical heterogeneity (Campopiano et al. 2018). Future research with EEG-IP can begin to systematically test new methods and validated processes across multiple samples. EEG-IP also fosters the potential for expanded and novel analytic techniques to infant data, such as connectivity, network, cluster, machine learning and harmonics analyses. Applying such techniques to infant data has traditionally been limited because signal-to-noise ratios are less well established than for older children. We expect EEG-IP to facilitate innovative solutions to these challenges, yielding new analytic tools and pipelines.

## Supplementary information

**Additional file 1: Figure S1.** Posterior channel region for supplementary analysis. Channels used as posterior cluster for ERP analysis. Channels in black were taken from selection used in Elsabbagh et al. (2012). Channels in grey were taken from selection used in Jones et al. (2016).

**Additional file 2: Figure S2.** Condition ERPs for full sample of merged dataset. Top: Grand average ERPs for face (dashed line) and non-face (solid line) stimuli. Topographical maps to the right show distribution of scalp voltages at the peak of the P400 response. Bottom: Bootstrapped difference wave and 95% confidence intervals for the face vs. non-face ERP effect.

**Additional file 3: Figure S3.** Condition ERPs and autism risk. Top: Grand average ERPs for face (dashed line) and non-face (solid line) stimuli, separated by low (red) and high (blue) risk infants. Topographical maps to the right show distribution of scalp voltages at the peak of the P400 response. Bottom: Bootstrapped difference wave and 95% confidence intervals for the condition (face/non-face) by risk (low/high) interaction.

**Additional file 4: Figure S4.** Condition ERPs and autism risk and outcome. Top: Grand average ERPs for face (dashed line) and non-face (solid line) stimuli, separated by low risk no ASD (red), high risk no ASD (blue), and high risk ASD (grey) infants. Topographical maps to the right show distribution of scalp voltages at the peak of the P400 response. Bottom: Bootstrapped difference wave and 95% confidence intervals for the condition (face/non-face) by risk (low/high) and outcome interaction.

**Additional file 5:** Supplementary Materials (Benjamini and Hochberg 1995; Ewen et al. 2019; Groppe et al. 2011; Key and Corbett 2019).

## Data Availability

The datasets generated during and/or analysed during the current study are not publicly available due the requirement for bilateral agreements between contributing sites. It is possible that the data will be made available publicly in the future, or from the corresponding author on reasonable request.

## Abbreviations

ASD: Autism Spectrum Disorder
BIDS-EEG: Brain Imaging Data Structure-EEG
EEG: Electroencephalography
EEG-IP: EEG-Integrated Platform
EGI: Electro-Geodesic Inc.
EMG: Electromyography
HED: Hierarchical Event Descriptor
HPC: High Performance Computing
ICA: Independent Component Analysis
ICs: Independent Components
