# Supplementary figures and images for: EEG-IP: an international infant EEG data integration platform for the study of risk and resilience in autism and related conditions
Source: Mol Med. 2020 May 7;26:40. doi: 10.1186/s10020-020-00149-3 (PMC7203847; doi:10.1186/s10020-020-00149-3)

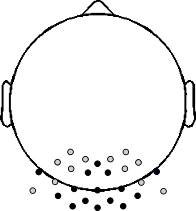

Supplement: Supplementary file 1 — Additional file 1: Figure S1. Posterior channel region for supplementary analysis. Channels used as posterior cluster for ERP analysis. Channels in black were taken from selection used in Elsabbagh et al. (2012). Channels in grey were taken from selection used in Jones et al. (2016). [file 10020_2020_149_MOESM1_ESM.png]

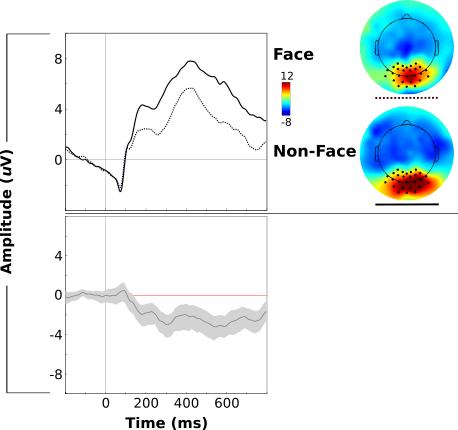

Supplement: Supplementary file 2 — Additional file 2: Figure S2. Condition ERPs for full sample of merged dataset. Top: Grand average ERPs for face (dashed line) and non-face (solid line) stimuli. Topographical maps to the right show distribution of scalp voltages at the peak of the P400 response. Bottom: Bootstrapped difference wave and 95% confidence intervals for the face vs. non-face ERP effect. [file 10020_2020_149_MOESM2_ESM.png]

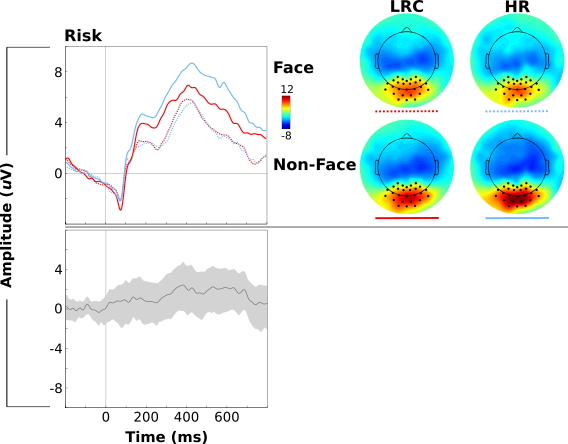

Supplement: Supplementary file 3 — Additional file 3: Figure S3. Condition ERPs and autism risk. Top: Grand average ERPs for face (dashed line) and non-face (solid line) stimuli, separated by low (red) and high (blue) risk infants. Topographical maps to the right show distribution of scalp voltages at the peak of the P400 response. Bottom: Bootstrapped difference wave and 95% confidence intervals for the condition (face/non-face) by risk (low/high) interaction. [file 10020_2020_149_MOESM3_ESM.png]

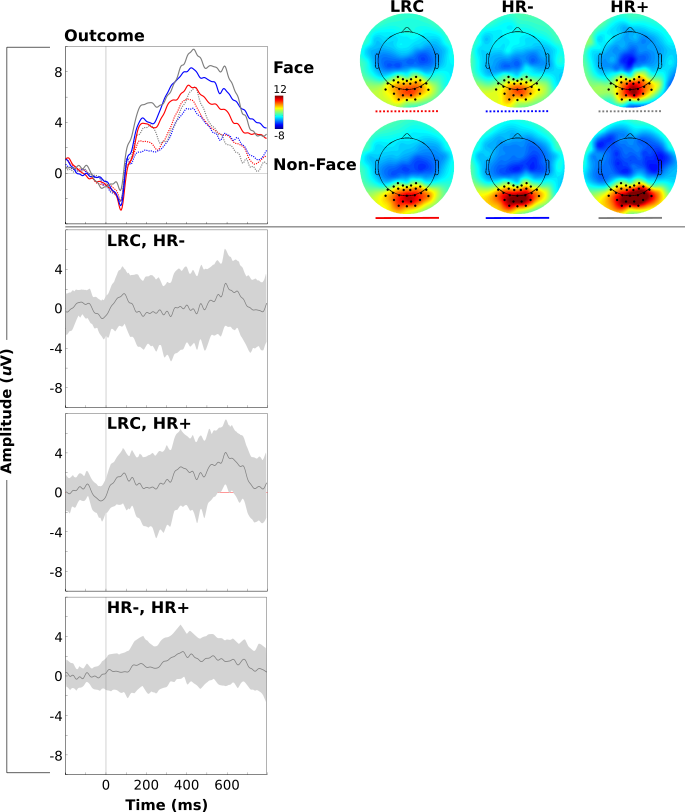

Supplement: Supplementary file 4 — Additional file 4: Figure S4. Condition ERPs and autism risk and outcome. Top: Grand average ERPs for face (dashed line) and non-face (solid line) stimuli, separated by low risk no ASD (red), high risk no ASD (blue), and high risk ASD (grey) infants. Topographical maps to the right show distribution of scalp voltages at the peak of the P400 response. Bottom: Bootstrapped difference wave and 95% confidence intervals for the condition (face/non-face) by risk (low/high) and outcome interaction. [file 10020_2020_149_MOESM4_ESM.png]
