# Supplementary material for: EEG-IP: an international infant EEG data integration platform for the study of risk and resilience in autism and related conditions
Source: Mol Med. 2020 May 7;26:40. doi: 10.1186/s10020-020-00149-3 (PMC7203847; doi:10.1186/s10020-020-00149-3)
Supplement: Supplementary file 5 — Additional file 5: Supplementary Materials (Benjamini and Hochberg 1995; Ewen et al. 2019; Groppe et al. 2011; Key and Corbett 2019). [file 10020_2020_149_MOESM5_ESM.doc]

## **Supplementary Materials**

#### *Data staging*

Following the initial loading and annotation of the data file the pipeline allows for the optional execution of a “staging” script. The staging script is intended to perform operations on the data that may be unique to the data acquisition project. In the case of the EEG-IP infant autism data the staging scripts are used to harmonize the data across recording sites and account for large durations of extreme artifacts in the recordings. The staging script first applies a transformation matrix that is specific to each channel location montage used across the projects and then applies a 1Hz high pass filter and a line noise notch filter (59Hz-61Hz for Boston and Washington sites and 49Hz-51Hz for the London site). These initial filters were used to equate the data across sites due to large variations found in initial diagnostics for power mains noise as well as low frequency contamination from movement artifacts.

After the filtering of the data the remaining procedures in the staging script are designed to address coarse artifacts in the data by assessing parameter estimates from all recording session combined in order to identify channels and periods of time within recordings that are outliers relative to the rest of the recording sessions in the data set. The goal of this process is to identify large artifacts in the data (e.g., damaged recording sites, long durations of large movement artifacts or equipment issues, etc.) such that these issues are flagged and then the remaining channels are used to calculate an average re-reference.

In the case of the EEG-IP infant datasets, there were no consistent event markers that could be used to isolate periods of time in the data that contained non-representational data. It was also determined through group level diagnostics that a 50µV standard deviation was a reliable parameter estimate for identifying one-second epoch outliers per channel. Further, this group-based outlier detection was suitable for more of the files if a 1Hz high-pass filter and a line-noise notch filter were applied during staging based on the group level outlier estimation. The full staging process for the EEG-IP data sets included an outlying channel identification, average re-referencing, 1Hz high pass, 59Hz-61Hz or 49Hz-51Hz notch filter, 50µV fixed threshold for classifying time and channel artifacts, and channel co-registration to a common head surface.

Once the filters are applied, the staging script identifies extremely bad channels before applying an average re-reference to the data. This initial average re-referencing of the data is used to equate the voltage value variances across channels within each file. Given that the data from these sites were collected with EGI systems, the raw data are referenced to the vertex site and thus have large differences in channel variance within each recording as a result of varying spatial proximity to the reference location. This reference-related voltage variance across channels impedes the detection of unusual channels by artificially increasing the range of voltage values across channels. Before re-referencing the data to the average site, an initial channel detection criteria is required to avoid including channels that dramatically influence the reference signal. Including a single channel with extreme voltages in the reference can contaminate all other channels to a degree that results in time periods being marked unnecessarily for rejection. For the selection of reference channels, the standard deviation of voltage values are calculated for each second of data in the recording. Then, for each time window, the voltage variance values across channels are transformed into distance values. The distance values are calculated as the values minus the median, divided by the inter-quantile (30% and 70%) range. The criteria for channel rejection for the initial average reference calculation is then assessed by calculating the average distance value across all time windows and identifying channels whose average distance is greater than the median plus six inter-quantile ranges (30% and 70%) of the average distance values across channels.

Many of the files in the EEG-IP repository contained long durations of large voltage artifacts, which is typical of infant movement behavior. Because the common criterion functions in the standardized trajectory of the Lossless artifact detection procedures use distributions of values within each data file, these large durations of non-representative artifacts make it difficult for the relative criteria measures to make accurate distinctions about unusual properties in the data. To correct for this in the staging process, study wide parameter estimates are calculated for variables that should be relatively constant across recording sessions. The absolute voltage values (calculated as the standard deviation of voltages in one-second time windows) is used as a constant parameter estimate to identify large durations of artifactual time based on properties across the sample of recording sessions. For this parameter estimate the channel criteria for rejection is calculated on each data file after applying a trimmed mean average reference as well as a 1Hz and line-noise notch filter. The channel voltage cutoff values are then calculated using the criterion function of 6 inter-quantile ranges (30% to median and median to 70%). The median cutoff value across channels is taken and then the median across session files is taken. Using this method, in combination with varying parameters, a global cutoff value of 50µV was selected as the fixed limit for the staging criterion function. The staging script applied this fixed criterion, calculating the standard deviation value for each channel in each one-second time interval, and then marks time windows in which 30% of the channels exceeded 50µV. Because this classification measure is intended to identify very large voltage artifacts, a one-second padding period is added to each side of identified time intervals to extend the duration of the annotation. The time periods that are marked as exceeding the fixed study parameter estimate criterion are stored in the *time_info* annotation structure with the label “*ch_s_sd*”. This method was then applied for marking channels by calculating the standard deviation value for each channel in each one-second time interval that was not already marked by the fixed criterion, and then marked channels in which 30% of the remaining time windows exceeded 50µV. The channels that are marked as exceeding the fixed study parameter estimate criteria are stored in the *chan_info* annotation structure with the label “*ch_s_sd*”. Because this fixed staging criterion does not consider the distributions within the recording session, but rather parameter estimates established across recording sessions, this method is capable of flagging very large or very small percentages of the data within a file as being different from norms across files.

#### *Face vs. non-face ERPs*

*EEG post-processing*

Post-processing for EEG-IP including purging channels, time, and independent components that were flagged as artifact during pre-processing and interactive quality control review. The remaining data were filtered with a low pass of 30Hz and epochs were generated around face and non-face stimuli (-200 to 800 ms) with a -200 to 0 ms baseline correction. Channels that had been removed were interpolated using spherical spline and all the data were re-referenced using the average of all channels. To merge the data across sites, analyses were carried using the average of a posterior channel cluster (see Figure S1), which included the channels used in Elsabbagh et al. (2012) and Jones et al. (2016). Descriptive summaries for sample size and trial count are presented in Supplementary Table 1.

**--- insert Figure S1 here ---**

**Supplementary Table 1.** Descriptive statistics for sample size and trial count by group and condition.

|  | **Face** | | **Non-face** | |
| --- | --- | --- | --- | --- |
|  | *n* | *M* (SD) | *n* | *M* (SD) |
| **LRC** | *75* | *29.88* (13.76) | *72* | *25.15* (10.61) |
| **HRA-** | *55* | *30.24* (14.97) | *56* | *23.36* (10.54) |
| **HRA+** | *26* | *28.96* (18.42) | *25* | *22.96* (13.10) |

*Statistical analysis*

The STATSLAB software package was used to implement robust ANOVAs, using percentile bootstrap, to examine differences between stimulus type, risk, and outcome status (Campopiano, van Noordt, & Segalowitz, 2018). Specifically, for each pair-wise contrast the single trial data from each condition were randomly re-sampled with replacement, averaged, and the difference wave was calculated. Repeating this process 1000 times generates a distribution of the average difference between conditions. Effects were compared against the 95% confidence of the bootstrapped differences. This procedure considers the full duration of effects as opposed to focusing solely on peak amplitude at specific latencies. Given that the full time course is used, we applied the Benjamini-Hochberg FDR (Bejmanini & Hochberg, 1995; Groppe et al., 2011) multiple comparison correction for each contrast to control family wise error rate.

*Results*

Although the focus of the current paper is not on post-processing strategies using data from EEG-IP, we did perform ERP analyses that allowed us to merge two independent data sets included in EEG-IP. Specifically, we pooled EEG recordings from 6-7 month infants taken from the London and Washington data sets as they contained a face processing task with both face and non-face stimuli. Previous findings from these independent samples, and other studies, find that visually evoked ERPs, particularly the P400, can reveal face processing dynamics in infants (Elsabbagh et al., 2012; Jones et al., 2016). For example, the P400 is found to be larger in amplitude to non-face compared to face stimuli at posterior electrodes (Elsabbagh et al., 2012; Jones et al., 2016). In addition, previous work examined risk and outcome, but failed to find evidence that P400 amplitude to faces were able to differentiate familial risk status or ASD outcome in high-risk infants. A complete description of the task parameters and channel selection for London and Washington can be found in Elsabbagh et al. (2012) and Jones et al. (2016), respectively. For our validation analyses, we combined London and Washington conditions for face and non-face stimuli and merged the channel clusters from both data sets to derive a region of interest over posterior electrodes (see Figure S1).

Our results replicate previous findings from the independent London and Washington samples. Similar to both Elsabbagh et al. (2012) and Jones et al. (2016), we observed a significant P400 effect that is larger to non-face than face stimuli (Figure S2). Similar to Elsabbagh et al. (2012) and Jones et al. (2016), we did not find evidence that the P400 between face and non-face stimuli varies as a function of risk status (Figure S3) or outcome (Figure S4). These findings, using a larger integrated data set, suggest that visual ERPs to face and non-face stimuli are not robust biomarkers that are sufficiently sensitive to differentiate autism risk or outcome. EEG-IP, and similar repositories, will be critical for validating biomarkers that are sensitive in differentiating risk, outcome, and potential treatment effects (Ewen, Sweeney, & Potter, 2019; Key & Corbett, 2019).

**--- insert Figure S2 here ---**

**--- insert Figure S3 here ---**

**--- insert Figure S4 here ---**
